# The Effect of Fatal Carbon Monoxide Poisoning on the Equilibria Between Cell Membranes and the Electrolyte Solution

**DOI:** 10.1007/s00232-014-9753-x

**Published:** 2014-11-22

**Authors:** Aneta D. Petelska, Joanna Kotyńska, Zbigniew A. Figaszewski

**Affiliations:** 1 Institute of Chemistry, University of Bialystok, Al. J. Pilsudskiego 11/4, 15-443 Białystok, Poland; 2 Laboratory of Electrochemical Power Sources Faculty of Chemistry, University of Warsaw, Pasteur St. 1, 02-093 Warsaw, Poland

**Keywords:** Acid–base equilibria, Surface charge density, Microelectrophoresis, Erythrocytes, Thrombocytes, Fatal carbon monoxide poisoning

## Abstract

The effect of fatal carbon monoxide poisoning on equilibria between cell membranes and surrounding ions was described using a theoretical four-equilibria model. The model was developed to obtain parameters characterizing the interactions between solution ions and erythrocyte or thrombocyte membrane surface. The parameters are the total surface concentrations of both acidic and basic groups *C*_A_, *C*_B_ and their association constants with solution ions *K*_AH_, *K*_BOH_. These parameters were used to calculate the theoretical values of surface charge density. The model was validated by comparison of these values to experimental data, which were determined from the electrophoretic mobility measurements of the blood cells. The experimental and theoretical surface charge density values agree at pH 2–8, and at higher pH, the deviation was observed.

## Introduction

The cell membrane is a fundamental for existence of each, living cells. All the biochemical processes, which take place inside the structures surrounded by a membrane and in a membrane, provide normal functioning of cells. However, the processes change significantly under the influence of various toxic factors as well as physiological and non-physiological conditions of the body. Many reports in the literature have shown changes in the structure and physicochemical properties of biological membranes, which are observed in physiological processes such as aging and leads to death as a consequence (Kala and Chudzikiewicz 2003) and in pathological, such as cardiovascular disease (Langlois 2009), tumors (Madej 1999; Szachowicz-Petelska et al. 2010), fatal carbon monoxide poisoning (Weaver 2009; Szeremeta et al. 2013).

Carbon monoxide (CO) gas is a product of the incomplete combustion of carbon-based fuels and substances. From a public health perspective, CO poisoning may be the cause of more than 50 % of fatal poisonings in many industrial countries (Omaye 2002). Exposure to higher concentrations of CO can result in death. The effects of carbon monoxide are largely the result of the formation of carboxyhemoglobin (COHb). The formation of COHb is a reversible process, but due to the affinity of carbon monoxide to hemoglobin, the elimination half-time varies, depending on the initial levels of COHb and the ventilation rate of the individuals. This might lead to accumulation of COHb, and even relatively low concentrations of carbon monoxide might produce substantial blood levels of COHb (Hauck and Neuberger 1984; Sedda and Rossi 2006). Coma, convulsions, cardiopulmonary arrest, and death may occur at COHb levels higher than 50 % (Sedda and Rossi 2006).

Any abnormalities in the functioning of the cells affect the physicochemical properties of the cell membrane, such as surface charge. Since this parameter depends on the composition of the membrane, any changes in the quantity and quality of charged functional groups on its surface cause changes in the surface charge values, thus affecting membrane–solution equilibria.

This work continues the systematic study of electrical properties of human erythrocytes and thrombocytes membranes realized by Figaszewski and co-workers (Petelska et al. 2012; Kotyńska et al. 2012; Szeremeta et al. 2013). In our previous paper (Szeremeta et al. 2013), we presented changes of the surface charge of blood cells after fatal carbon monoxide poisoning obtained only on the experimental way. The experiment was performed using microelectrophoresis method, which is one of the basic analytical tools for biological studies. Due to the lack of literature data concerning the effect of exogenous carbon monoxide on equilibria between cell membranes and surrounding ions, in this paper, we proposed a theoretical model describing the equilibria. The model was validated by comparing with experimental data. In our opinion, the quantitative description of cell membrane properties can help in better understanding the effect of fatal carbon monoxide poisoning on biological membrane surface properties.

## Theory

Dependence of surface charge density of cell membrane on the pH of electrolyte solution can be described with help of the four-equilibrium model. The mathematical formulation of this model was proposed by Dobrzyńska and co-workers and presented in full details in the papers elsewhere (Dobrzyńska et al. 2006; Kotyńska et al. 2012). Assumptions of the model are described by Eqs. (1)–(7). The electrolyte solution ions (H^+^, OH^−^, Na^+^, Cl^−^) are adsorbed at the cell membranes (erythrocytes or thrombocytes). Two equilibria are connected with positive groups (for example: phospholipids or proteins and sodium and hydrogen ions), and two concern the negative species of phospholipids or proteins and hydroxide and chloride ions. The adsorption equilibria (Eq. 1–4) can be presented in the form:

1 $${\text{A}}^{ - } + {\text{ H}}^{ + } \Leftrightarrow {\text{AH,}}$$

2 $${\text{A}}^{ - } + {\text{ Na}}^{ + } \Leftrightarrow {\text{ANa,}}$$

3 $${\text{B}}^{ + } + {\text{ OH}}^{ - } \Leftrightarrow {\text{BOH,}}$$

4 $${\text{B}}^{ + } + {\text{ Cl}}^{ - } \Leftrightarrow {\text{BCl,}}$$

5 $$C_{\text{A}} = a_{{{\text{A}}^{ - } }} + a_{\text{AH}} + a_{\text{ANa}} ,$$

6 $$C_{\text{B}} = a_{{{\text{B}}^{ + } }} + a_{\text{BOH}} + a_{\text{BCl}} ,$$

7 $$\delta = (a_{{{\text{B}}^{ + } }} - a_{{{\text{A}}^{ - } }} ) \cdot F,$$

where $a_{\text{AH}}$, $a_{\text{ANa}}$, $a_{{{\text{A}}^{ - } }}$, $a_{\text{BOH}}$, $a_{\text{BCl}}$, and $a_{{{\text{B}}^{ + } }}$ are surface concentrations of corresponding groups on the membrane surface; $a_{{{\text{H}}^{ + } }}$, $a_{{{\text{Na}}^{ + } }}$, $a_{{{\text{OH}}^{ - } }}$, and $a_{{{\text{Cl}}^{ - } }}$ are volume concentrations of solution ions; $C_{\text{A}}$is the total surface concentration of the membrane acidic groups; $C_{\text{B}}$ is the total surface concentration of the membrane basic groups, $F = 96487$ $\left[ {\frac{C}{\text{mol}}} \right]$ is Faraday constant; and $\delta$ is the surface charge density.

Final equations (Dobrzyńska et al. 2006; Kotyńska et al. 2012):

- equation describing surface charge density of the cell membrane:
  8 $$\frac{\delta }{F} = \frac{{C_{\text{B}} }}{{1 + K_{\text{BOH}} a_{{{\text{OH}}^{ - } }} + K_{\text{BCl}} a_{{{\text{Cl}}^{ - } }} }} - \frac{{C_{\text{A}} }}{{1 + K_{\text{AH}} a_{{{\text{H}}^{ + } }} + K_{\text{ANa}} a_{{{\text{Na}}^{ + } }} }}.$$
- linear equations obtained by simplification of Eq. (8), valid for high (Eq. 9) and low (Eq. 10) concentration of hydrogen ions:
  9 $$\frac{{\delta a_{{{\text{H}}^{ + } }} }}{F} = \frac{{C_{\text{B}} }}{{1 + K_{\text{BCl}} a_{{{\text{Cl}}^{ - } }} }}a_{{{\text{H}}^{ + } }} - \left( {\frac{{C_{\text{B}} K_{\text{BOH}} K_{\text{W}} }}{{(1 + K_{\text{BCl}} a_{{{\text{Cl}}^{ - } }} )^{2} }} + \frac{{C_{\text{A}} }}{{K_{\text{AH}} }}} \right),$$
  10 $$\frac{\delta }{{Fa_{{{\text{H}}^{ + } }} }} = - \left( {\frac{{C_{\text{A}} }}{{1 + K_{\text{ANa}} a_{{{\text{Na}}^{ + } }} }}} \right)\frac{1}{{a_{{{\text{H}}^{ + } }} }} + \left( {\frac{{C_{\text{B}} }}{{K_{\text{BOH}} K_{\text{W}} }} + \frac{{C_{\text{A}} K_{\text{AH}} }}{{(1 + K_{\text{ANa}} a_{{{\text{Na}}^{ + } }} )^{2} }}} \right),$$

where $K_{\text{AH}}$, $K_{\text{ANa}}$, $K_{\text{BOH}}$, and $K_{\text{BCl}}$ are association constants.

The coefficients describing these linear functions may be easily obtained using linear regression and subsequently applied to calculate the parameters. The calculation of the following parameters $C_{\text{A}}$, $C_{\text{B}}$, $K_{\text{AH}}$, $K_{\text{BOH}}$ is possible owing to knowledge of the association constants -$K_{\text{ANa}}$, $K_{\text{BCl}}$ obtained for phosphatidylcholine liposome membrane (Dobrzyńska et al. 2007). Defining the values of these parameters permits the calculation of the theoretical cell membrane surface charge from Eq. (8) enabling comparison to experimental data.

## Materials and Methods

### Materials

Approval for this study was granted by the Ethics Review Board of Medical University of Bialystok (No. R-I-002/533/2010). Blood (pH ~6.8) was obtained from sober individuals during autopsies made at the Forensic Medicine Department at the Medical University of Bialystok. The subject of the examination was based on 10 selective fatal carbon monoxide poisonings in fire (6 men and 4 women; mean age 43.4 years, range 23–63) autopsied in the year 2010–2011. Blood was routinely obtained from the femoral vein and put into chemically and biologically cleaned glass containers and was donated to the Department of Electrochemistry at the University of Bialystok. The donated samples were comparatively analyzed to the control samples taken from live individuals from the Blood-service Centre in Bialystok.

#### Preparation of Erythrocytes from Blood

Erythrocytes were isolated from 2 ml of liquid whole blood by centrifugation at 900×*g* for 8 min at room temperature. The supernatant thrombocyte-rich plasma was removed and saved for subsequent processing while the erythrocytes were washed three times with isotonic saline (0.9 % NaCl) at 3,000×*g* for 15 min. After the final wash, the erythrocyte pellet was resuspended in isotonic saline for electrophoretic measurement.

#### Preparation of Thrombocytes from Plasma

The thrombocyte-rich plasma was centrifuged at 4,000×*g* for 8 min. The supernatant plasma was removed and discarded. The thrombocyte pellet was washed three times with isotonic saline by centrifugation at 3,000×*g* for 15 min. After the final wash, the thrombocytes were resuspended in isotonic saline for electrophoretic measurement

All solutions and cleaning procedures were performed with water purified using a Milli-Q system (18.2, Millipore, USA).

### Methods

#### Microelectrophoretic Mobility Measurements

The electrophoretic mobility of erythrocytes or thrombocytes vesicles in suspension was measured using laser doppler velocimetry (LDV) and a Zetasizer Nano ZS (Malvern Instruments, UK) apparatus. The measurements were carried out as a function of pH. The cell membranes were suspended in NaCl solution and titrated to the desired pH using HCl or NaOH. The reported values represent the average of at least six measurements performed at a given pH.

From electrophoretic mobility measurements, the surface charge density was determined using the Eq. (11) presented below (Alexander and Johnson 1949):

11 $$\updelta = \frac{\eta \cdot u}{d},$$

where *η* is the viscosity of solution, *u* is the electrophoretic mobility, *d* is the diffuse layer thickness. The diffuse layer thickness (Barrow 1996) was determined from the formula (Eq. 12):

12 $$d = \sqrt {\frac{{\varepsilon \cdot \varepsilon_{0} \cdot R \cdot T}}{{2 \cdot F^{2} \cdot I}}} ,$$

where *R* is the gas constant, *T* is temperature, *F* is the Faraday number, *I* is the ionic strength of 0.9 % NaCl, and *εε*_o_ is the permeability of the electric medium.

## Results and Discussion

**Fig. 1:**
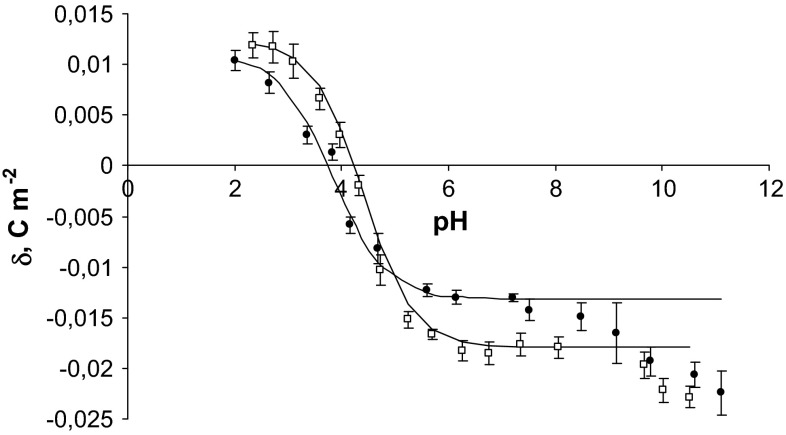
pH dependence of surface charge density of erythrocytes: *filled circle* control, *unfilled square* fatal carbon monoxide poisoning (the experimental values are indicated by *points* and the theoretical values by the *curve*)

**Fig. 2:**
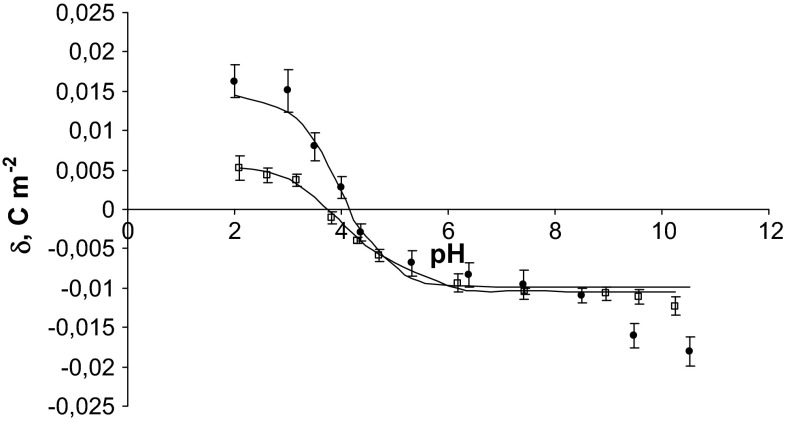
pH dependence of surface charge density of thrombocytes: *filled circle* control, *unfilled square* fatal carbon monoxide poisoning (the experimental values are indicated by *points* and the theoretical values by the *curve*)

The effect of fatal carbon monoxide poisoning on equilibria between cell membranes and surrounding ions was described. The experimental data of surface charge density were calculated from measured electrophoretic mobility values using Eq. (11). The measurements were performed at several pH values using 0.155 M NaCl as a supporting electrolyte. The theoretical values of surface charge density were determined by applying Eq. (8) to the experimental data. Experimental and theoretical surface charge density values of the erythrocyte and thrombocyte membranes as a function of pH are presented in Figs. 1 and 2, respectively. The experimental data are indicated by points, and the theoretical data are indicated by the curve.

Complexity of the biological membrane structure determines about equilibria occurring between the membrane components, as well as between them and surroundings. It is not possible to define values of each parameters of such a large number of equilibria. Therefore, it is necessary to assume an adequate number of parameters that would include the mean values for all equilibria. These parameters may be total surface concentration of membrane acidic and basic groups on a membrane surface or averaged association constants with solution ions. Determination of numerical values of such parameters is very important as it can help in better understanding and describing of changes in natural membranes, being a result of various factors and processes. The quantitative description of the interactions occurring between the cell membrane and surroundings was proposed by Dobrzynska et al. (2006) (presented in “Theory” section). The model enabled to evaluation of the membranes characterizing parameters—the total concentrations of functional acidic ($c_{\text{A}}$) and basic ($c_{\text{B}}$) groups on the erythrocyte as well as thrombocyte surface and their average association constants with hydrogen ($K_{\text{AH}}$) and hydroxyl ($K_{\text{BOH}}$) ions. These parameters were calculated based on Eqs. (9) and (10). The determination of all above parameters was feasible by making an assumption that the $K_{\text{ANa}}$ and $K_{\text{BCl}}$ association constant values are the same as those obtained for phosphatidylcholine liposomes. The $K_{\text{ANa}}$ and $K_{\text{BCl}}$ values of phosphatidylcholine surface groups with sodium and chloride ions were previously reported and are equal 0.230 and 0.076 (m^3^/mol), respectively (Dobrzyńska et al. 2007). The obtained $c_{\text{A}}$, $c_{\text{B}}$, $K_{\text{AH}},$ and $K_{\text{BOH}}$ values were substituted into Eq. (11) to produce a surface charge density vs pH theoretical curves for the studied cell membranes. The parameters characterizing both erythrocyte and thrombocyte surfaces are presented in Tables 1 and 2, respectively. These data were analyzed using standard statistical analysis and are expressed as mean standard deviation.

**Table 1:** The total concentrations of acidic and basic functional groups of erythrocytes and their association constants with H^+^ and OH^−^ ions

Groups	Parameters			
	*c*_A_ (10^−6^ mol/m^2^)	*c*_B_ (10^−6^ mol/m^2^)	*K*_AH_ (10^2^ m^3^/mol)	*K*_BOH_ (10^7^ m^3^/mol)
Control (Kotyńska et al. 2012)	7.06 ± 0.42	1.54 ± 0.47	3.39 ± 1.12	3.65 ± 0.84
Fatal carbon monoxide poisoning	5.22 ± 0.11	1.26 ± 0.09	5.61 ± 0.51	1.95 ± 0.29

**Table 2:** The total concentrations of acidic and basic functional groups of thrombocytes and their association constants with H^+^ and OH^−^ ions

Groups	Parameters			
	*c*_A_ (10^−6^ mol/m^2^)	*c*_B_ (10^−6^ mol/m^2^)	*K*_AH_ (10^2^ m^3^/mol)	*K*_BOH_ (10^7^ m^3^/mol)
Control (Kotyńska et al. 2012)	3.67 ± 0.79	1.17 ± 0.21	2.81 ± 1.70	2.04 ± 0.59
Fatal carbon monoxide poisoning	5.03 ± 0.21	0.56 ± 0.08	1.62 ± 0.26	1.06 ± 0.33

On analyzing the curves shown in Figs. 1 and 2, it can be noted that there is agreement between theoretical and experimental values in the pH range 2–8, and above pH > 8, the theoretical curves differ from the experimental points. The observed deviation at high pH values may be caused by the interactions occurring between the functional groups of the blood cells membrane components. The proposed mathematical model considers only the equilibria between membrane surface and the surrounding ions.

As it can be seen from Tables 1 and 2, the effect of fatal carbon monoxide poisoning on erythrocyte as well as thrombocyte membranes causes changes in the parameters values ($c_{\text{A}}$, $c_{\text{B}}$, $K_{\text{AH}}$, and $K_{\text{BOH}}$). $c_{\text{A}}$ of erythrocyte membranes decreased compared with control group; however, in the case of $c_{\text{B}}$, there were no statistically significant changes. *K*_AH_ increased, whereas *K*_BOH_ decreased compared with the control group (Table 1). $c_{\text{A}}$ of thrombocyte membranes increased, whereas $c_{\text{B}}$ decreased compared with control group. *K*_AH_ and *K*_BOH_ decreased compared with the control group (Table 2).

One of the most important elements of four-equilibria model is evaluation of membrane changes in blood cells. The observed changes in the total surface concentration of functional acidic and basic groups of blood cell membranes and their association constants with the electrolyte ions as a result of fatal carbon monoxide poisoning are likely caused by a number of various phenomena that occur in the human body after death. The decay of the natural structures of the body leads to a number of new as well as existing chemical compounds (Kala and Chudzikiewicz 2003). Death causes the change in blood pH; more than 60 years ago, it was discovered an increase in hydrogen ion concentration in tissues and body fluids (Straumfjord and Butler 1957). Later studies seemed to confirm these observations; Sawyer et al. (1988) demonstrated a sudden decrease in the pH of human blood at the time; within 24 h after death, the pH value was around 5.2. The normal blood pH is tightly regulated between 7.35 and 7.45. Deviation from these values indicates changes in acid–base equilibria occurring in biological membranes, which affects the electric charge on the membrane surface. Surface charge also strongly depends on molecular composition of the cell membrane, particularly of the type and number of surface functional groups. Numerous experiments studying such relationships were performed, by Figaszewski and co-workers, on model (Dobrzyńska et al. 2007; Kotyńska et al. 2008) as well as biological membranes (Szachowicz-Petelska et al. 2008, 2014; Petelska et al. 2012).

The theoretical analysis presented in this paper based on the four-equilibria model demonstrates the effect of fatal carbon monoxide poisoning on the electrical properties of blood cells membranes. We suppose that existing interactions between the cell membrane components and between them and surroundings lead to appearance of new functional groups on the membranes surfaces and/or to disappearance of the existing ones which in turn causes alterations in all analyzed parameters characterizing the cell membrane. Quantitative evaluation of the equilibria in our opinion is essential for the interpretation and understanding of a number of physical and chemical processes occurring in the human body after death. Calculated parameters (*C*_A_, *C*_B_, *K*_AH_, and *K*_BOH_) as well as the observed dynamics of surface charge changes may be helpful, for example, in the description of the pathophysiology of carbon monoxide poisoning deaths. It should be noted that our theoretical considerations are innovative. Despite the fact that presented studies are quite preliminary, there is no doubt that there is a necessity of their continuation toward obtaining information that may be helpful in solving a number of problems facing the forensic medicine. More in-depth research will provide essential information for forensic medicine.
